# De novo activating GUCY2C variant with prenatal bowel dilatation, neonatal functional obstruction, and reversible intestinal failure: A case report

**DOI:** 10.1016/j.intf.2026.100376

**Published:** 2026-06-11

**Authors:** Michele Maria Cantagalli, Tommaso Amato, Sofia Chioccioli, Riccardo Coletta, Antonino Morabito

**Affiliations:** a Department of Neurosciences, Psychology, Drug Research and Child Health (NEUROFARBA), University of Florence, Florence, Italy; b Department of Pediatric Surgery, Meyer Children’s Hospital, Florence, Italy

**Keywords:** GUCY2C, Congenital secretory diarrhea, Intestinal failure, Ileostomy, Ganglionitis, Intestinal rehabilitation

## Abstract

**Background:**

Activating variants in GUCY2C increase cyclic GMP signaling in enterocytes, driving chloride rich luminal secretion and impaired sodium absorption. Affected neonates can present with prenatal bowel dilatation and severe postnatal fluid losses that mimic mechanical obstruction.

**Case report:**

We describe a term male infant with fetal ultrasound and MRI evidence of markedly dilated bowel loops and severe polyhydramnios. Fetal testing identified a de novo heterozygous GUCY2C missense variant (c.2732 A>G). After birth, profuse intestinal secretion combined with profound intestinal hypotonia led to acute functional obstruction and massive ileal dilatation, prompting urgent laparotomy and diverting ileostomy. No mechanical stenosis was found; the ileocecal valve and colon were patent. Histology showed eosinophilic enteritis with prominent ganglionitis and preserved ganglion cells. The infant required prolonged parenteral nutrition with gradual advancement of enteral feeds and fecal recycling through the distal mucous fistula to achieve enteral autonomy by 2 years of age. He is currently 3 years old.

**Conclusion:**

GUCY2C mediated secretory diarrhea may be complicated by severe dysmotility and inflammatory enteric neuropathy, requiring early decompression and prolonged intestinal rehabilitation before recovery.

## Introduction

Congenital secretory diarrheas are rare disorders characterized by high volume watery intestinal losses beginning in the perinatal period [1], [2], [3]. Gain of function variants in GUCY2C, encoding the intestinal receptor guanylate cyclase C, increase intracellular cyclic GMP and stimulate chloride and water secretion while reducing sodium absorption [4], [5].

Prenatal bowel dilatation and polyhydramnios are frequent and can be misinterpreted as intestinal atresia. We report a neonate with a de novo GUCY2C variant whose early course was dominated not only by secretion but also by profound intestinal hypotonia with functional obstruction, followed by a prolonged yet reversible intestinal failure trajectory.

Congenital secretory diarrheas are rare disorders characterized by high volume watery intestinal losses that begin in the perinatal period and may rapidly lead to dehydration, electrolyte derangement, and intestinal failure [1], [2]. Among these conditions, activating variants in *GUCY2C*, which encodes the intestinal receptor guanylate cyclase C, increase intracellular cyclic GMP signaling, stimulate chloride and water secretion, and reduce sodium absorption [4], [5]. Clinically, affected infants may present before birth with polyhydramnios and bowel dilatation, while postnatally they often develop severe secretory diarrhea, salt wasting, and dependence on parenteral support [1], [2], [3].

Although the secretory phenotype of *GUCY2C* related disease is increasingly recognized, less attention has been paid to the possibility that, in some patients, intestinal dysmotility may substantially shape the early clinical course. In particular, marked prenatal and postnatal bowel dilatation may mimic a mechanical obstruction, complicating diagnosis and management and potentially prompting surgical exploration. Moreover, the extent to which secondary intestinal dysfunction, including inflammatory changes involving the enteric nervous system, contributes to neonatal intestinal failure in this setting remains insufficiently characterized.

Here, we report a term male infant with a de novo activating *GUCY2C* variant who presented with severe fetal bowel dilatation, neonatal functional obstruction, and prolonged but reversible intestinal failure. The case is clinically informative for three reasons. First, it illustrates how *GUCY2C* disease may present not only as secretory diarrhea but also as profound intestinal hypotonia with functional obstruction in the absence of an anatomic lesion. Second, it documents eosinophilic enteritis with prominent ganglionitis and preserved ganglion cells, raising the possibility that inflammatory enteric neuropathy contributed to the severity of dysmotility. Third, it highlights a successful intestinal rehabilitation pathway, including fecal recycling through the stoma, gradual advancement of enteral feeds, and eventual achievement of enteral autonomy. By emphasizing these features, we aim to broaden the clinical understanding of severe neonatal presentations associated with activating *GUCY2C* variants and to draw attention to practical management issues that may influence outcomes.

## Case report

### Prenatal period

At 12 weeks plus 3 days of gestation, ultrasound showed a dilated fetal bowel loop (maximum 4 mm). Villocentesis at 13 weeks plus 6 days showed a normal karyotype, normal array CGH, and negative testing for cystic fibrosis. A detailed anatomic scan at 17 weeks plus 1 day confirmed progressive bowel dilatation (maximum 19.4 by 5.9 mm). Fetal MRI at 20 weeks plus 1 day suggested a distal mid gut obstructive pattern and did not visualize colon or rectum. A full bowel genetic panel at 21 weeks plus 6 days identified a de novo heterozygous GUCY2C missense variant (c.2732 A>G), interpreted as likely pathogenic. Severe polyhydramnios developed (amniotic fluid index 46.2 cm at 29 weeks plus 2 days), requiring amnioreduction and antenatal corticosteroids.

### Postnatal presentation and surgical management

**Fig. 1:**
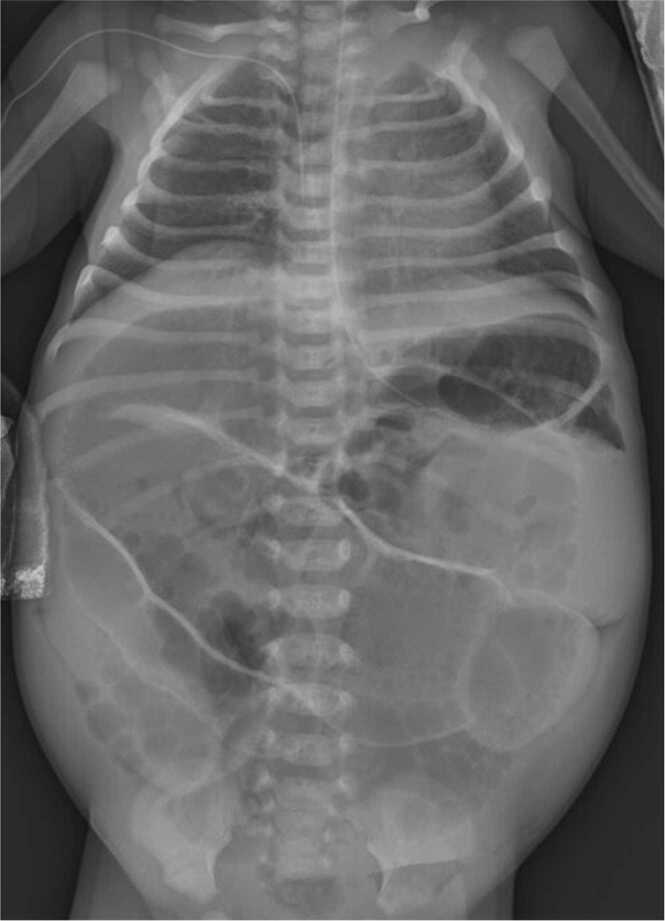
Bowel dilatation after birth.

Delivery was induced at 38 weeks plus 6 days. Birth weight was 3.06 kg and Apgar scores were 10 at 1 min and 10 at 5 min. Soon after birth, abdominal distension worsened with clinical deterioration (Fig. 1). Given the extreme bowel dilatation and concern for cardio respiratory compromise, urgent exploratory laparotomy was performed. Intraoperatively, the ileum was massively dilated and globally flaccid, with a caliber change beginning approximately 30 cm proximal to the ileocecal valve and extending proximally for roughly 50 cm. No intrinsic stenosis or obstructing lesion was identified. The ileocecal valve and colon were patent. A diverting ileostomy was created to decompress the small bowel and two ileal samples were taken near the caliber change and at the stoma sites.

### Histology

**Fig. 2:**
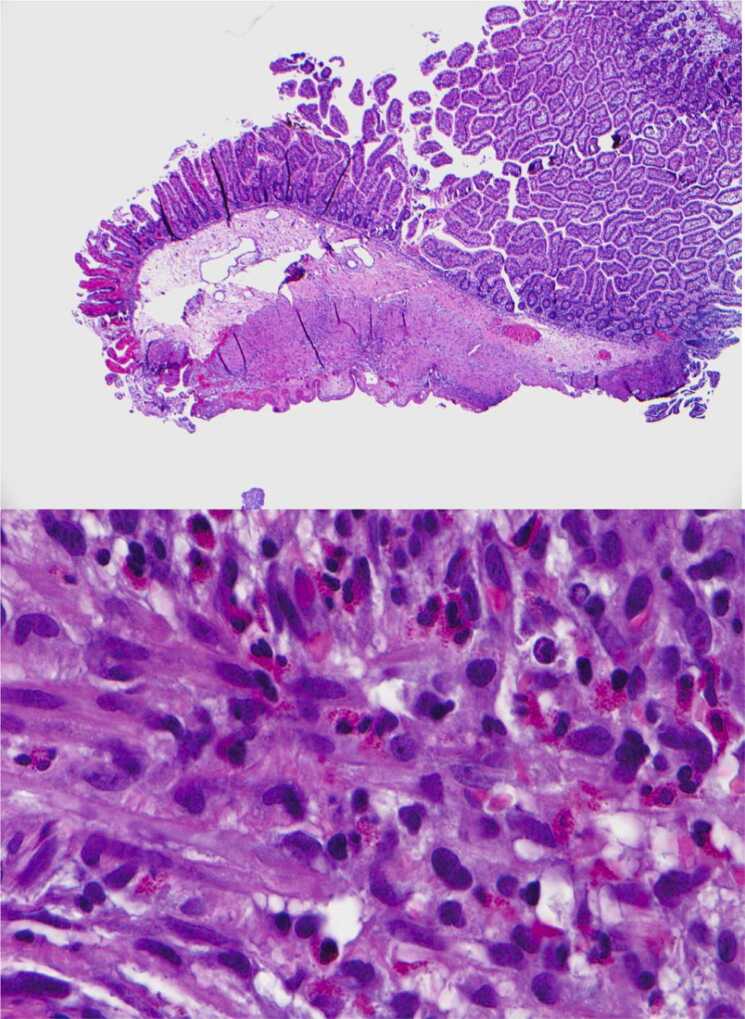
Sections of intestinal wall with a mucosal lining showing villi with preserved, architecturally normal features, without an increase in the inflammatory component (fewer than 25 lymphocytes per 100 epithelial cells). Within the lamina propria, glandular structures show features of hyperplasia and an increased inflammatory infiltrate in which eosinophilic granulocytes are prominent (20–30 per 40 × high power field). The ganglion cell component of the submucosal and myenteric nerve plexuses is normal.

Microscopy demonstrated eosinophilic enteritis with prominent ganglionitis. The submucosal and myenteric plexuses contained a normal complement of ganglion cells, arguing against aganglionosis (Fig. 2).

### Intestinal failure course and nutritional rehabilitation

**Fig. 3:**
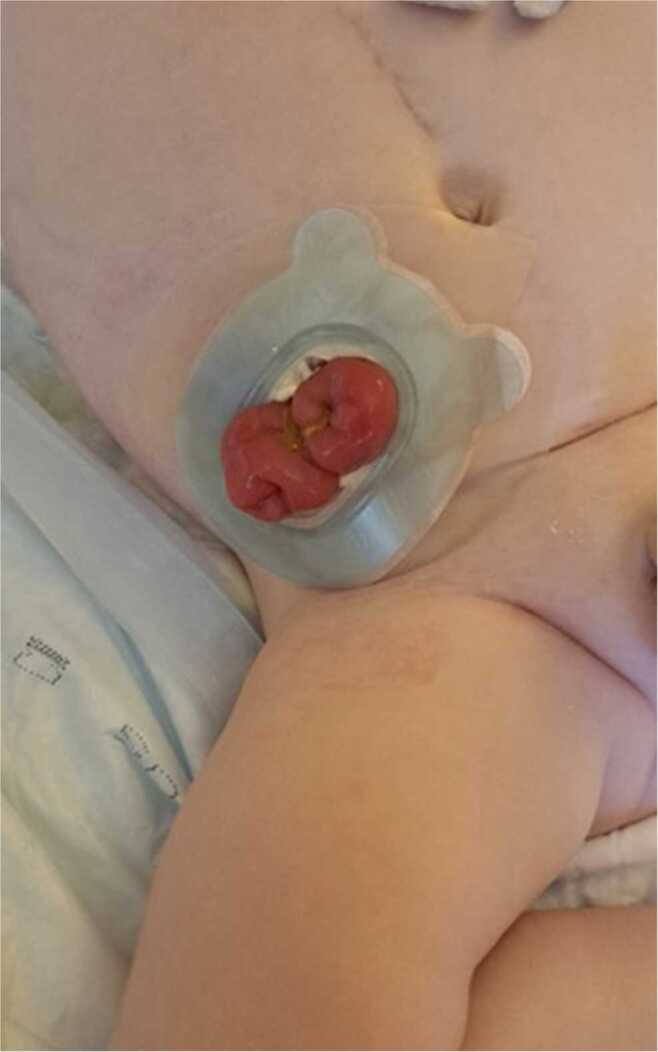
Stoma and pump system for fecal recycling.

**Fig. 4:**
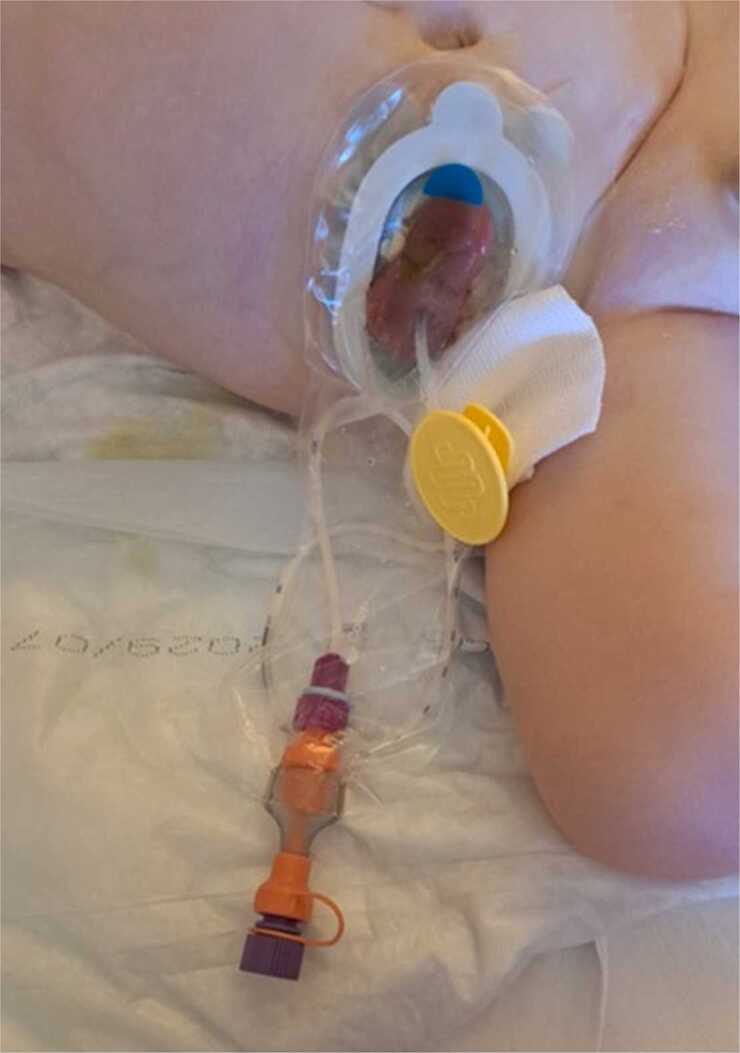
Stoma and pump system for fecal recycling.

The postoperative course was complicated by high output from the ileostomy and recurrent risk of dehydration with substantial sodium replacement needs, necessitating parenteral nutrition for growth and metabolic stability. Enteral feeding was advanced cautiously while parenteral calories were tapered in a stepwise manner. Key nutrition milestones, including the introduction of infant formula and complementary feeding, are summarized in Table 1. A key step to achieve enteral autonomy was the introduction after every evacuation of fecal recycling performed using a pump that collected fecal output from the stoma bag and delivered it into the distal fistula via a tube. (Fig. 3, Fig. 4)

**Table 1:** Nutrition milestones and growth during intestinal rehabilitation.

**Postnatal age (days)**	**Weight (g)**	**Enteral calories (kcal/kg/ day)**	**Parenteral calories (kcal/kg/day)**	**Total calories (kcal/day)**	**EN %**	**Milestone**
0	3060					Birth at 38 weeks plus 6 days. Early abdominal distension with functional obstruction. Urgent ileostomy and decompression.
108	6055	54	55	656.4	49.7%	Start of fecal recycling with detailed nutrition and output monitoring in database.
143	6800	58	36	633.2	61.8%	Enteral feeds advanced while parenteral calories remained substantial.
165	6800	45	36	547.6	56%	Infant formula introduced.
183	7620	70	22	700.8	77%	Complementary feeding initiated.
217	8520	95	20	974.0	83%	Marked tapering of parenteral calories with higher enteral intake.
243	9010	101	0.0	910.8	100%	Sustained parenteral independence achieved.
263	9150	125	0.0	1143.9	100%	Follow up with full enteral autonomy and continued growth.

**Fig. 5:**
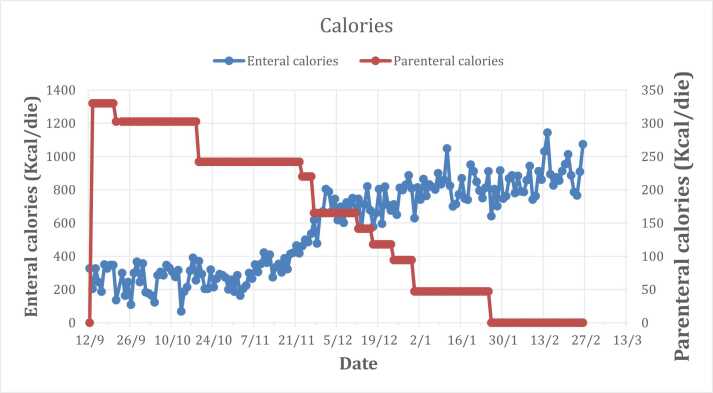
Enteral and parenteral calories over time. Daily enteral calories (blue) and parenteral nutrition calories (orange) across the monitored period, illustrating progressive enteral advancement and stepwise tapering of parenteral calories to sustained discontinuation.

**Fig. 6:**
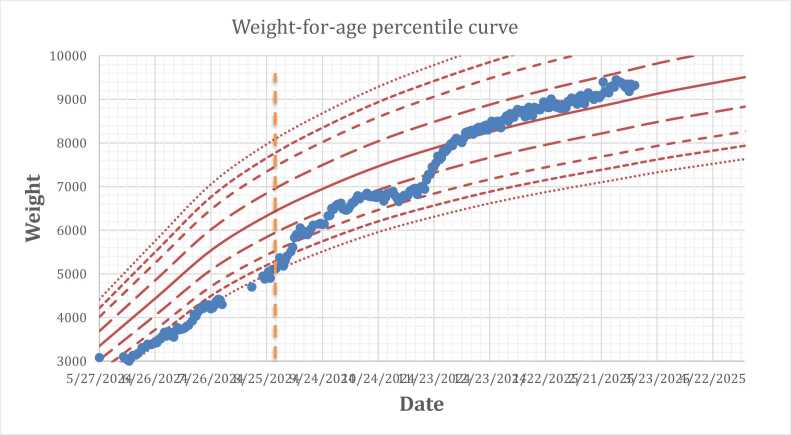
Weight-for-age percentile curves. Weight-for-age percentile curve. The dashed line corresponds to the start of fecal recycling.

Fig. 5 illustrates the transition from parenteral to enteral calories over time, with sustained parenteral independence achieved. Fig. 6 illustrates the weight increase in a weight-for-age percentile curve. At follow up, the infant maintained enteral autonomy with continued growth.

## Discussion

This case underscores three points relevant to clinicians managing neonatal intestinal failure. First, prenatal bowel dilatation in GUCY2C disease can closely resemble mechanical obstruction. This diagnostic ambiguity is well documented and has led to unnecessary or repeated surgical explorations in prior reports [1], [2], [6].

In this patient, fetal genetic confirmation supported anticipatory planning for severe postnatal fluid and electrolyte losses. Second, beyond secretion, the early course was dominated by severe dysmotility and functional obstruction with extreme ileal dilatation, prompting urgent decompressive ileostomy despite the absence of an anatomic blockage. Similar patterns have been described in congenital sodium diarrhea where fluid filled dilated bowel and apparent obstruction precipitated early diversion [6].

More broadly, intestinal pseudo-obstruction can arise from inflammatory neuropathies, including eosinophilic myenteric ganglionitis, a rare but highly morbid entity that has been reported even in neonates and can mimic Hirschsprung disease while preserving ganglion cells [7], [8].

The presence of prominent ganglionitis with intact ganglion cells in our case supports an inflammatory enteric neuropathy as a plausible contributor to the profound hypotonia and inability to clear secreted luminal fluid. Whether this inflammatory neuropathy is secondary to sustained mucosal injury from secretory burden, coincidental, or mechanistically linked to altered GC C signaling remains unknown. Experimental and clinical observations suggest GC C signaling has broader effects on intestinal homeostasis and may interact with inflammatory pathways in a subset of patients [4], [5].

Third, the nutritional trajectory emphasizes that intestinal failure in this setting can be reversible with structured intestinal rehabilitation. The child required prolonged parenteral support during the period of maximal ostomy losses and limited absorptive reserve but achieved enteral autonomy after gradual advancement of enteral calories, fecal recycling and careful weaning of parenteral nutrition. This pattern aligns with the central goals of pediatric intestinal rehabilitation: maintain growth and hydration, minimize catheter exposure, and transition toward enteral autonomy when the clinical course permits.

From a management standpoint, the case reinforces practical implications for multidisciplinary care. Prenatal recognition of a secretory diarrhea gene can help counsel families, prepare early electrolyte management, and reduce reflexive assumptions of atresia. After birth, persistent distension and clinical deterioration should prompt consideration of functional obstruction in addition to secretion, especially when luminal fluid production is extreme. Finally, histologic evaluation of enteric nervous system inflammation is important when dysmotility is disproportionate, as eosinophilic ganglionitis may represent a treatable contributor in selected cases, although optimal therapy is not established and evidence is limited to case-based experience [7], [8].

## Conclusion

This case broadens the clinical understanding of activating GUCY2C variants by demonstrating that secretory diarrhea may be exacerbated by severe intestinal dysmotility and inflammatory enteric neuropathy, leading to acute functional obstruction and prolonged neonatal intestinal failure. In this context, prenatal bowel dilatation can closely resemble mechanical obstruction, highlighting the importance of early genetic diagnosis to inform anticipatory management and prevent misinterpretation of imaging results. Postnatally, the combination of excessive luminal secretion and impaired motility may require early decompressive diversion, even in the absence of anatomical lesion. Notably, this case demonstrates that intestinal failure associated with CUCY2C activation can be reversible. With prompt surgical decompression, careful rehabilitation, including fecal recycling, full enteral autonomy and sustained growth are achievable. Identifying dysmotility and enteric nervous system inflammation as potential factors in severe cases may enhance both diagnostic assessment and individualized management strategies for affected neonates.

## CRediT authorship contribution statement

**Antonino Morabito:** Writing – review & editing, Visualization, Validation, Supervision. **Riccardo Coletta:** Visualization, Validation, Supervision. **Sofia Chioccioli:** Visualization, Validation, Resources. **Tommaso Amato:** Software, Resources, Investigation. **Michele Maria Cantagalli:** Writing – original draft, Project administration, Methodology, Investigation, Formal analysis, Data curation, Conceptualization.

## Patient's or guardian's consent

Written informed consent for publication of clinical details and figures was obtained from the patient's legal guardians.

## Ethical statement

This case report was conducted in accordance with the principles of the Declaration of Helsinki. Written informed consent was obtained from the patient for publication of this case report and any accompanying images. Ethics committee approval was not required for this study, as it is a retrospective report of a single clinical case. All identifying information has been removed to protect patient confidentiality.

## Funding

This research did not receive any specific grant from funding agencies in the public, commercial or not for profit sectors.

## Declaration of Competing Interest

None.

## References

[1] Fiskerstrand T., Arshad N., Haukanes B.I. (2012). Familial diarrhea syndrome caused by an activating mutation in GUCY2C. N Engl J Med.

[2] Muller T., Rasool I., Heinz E. (2015). Congenital secretory diarrhea caused by activating germline mutations in GUCY2C. Gut.

[3] Yuan C., Lyu J., Sun X., Wu J., Liu Y. (2026). Congenital diarrhea and enteropathies caused by a heterozygous mutation in the GUCY2C gene: a rare case report. Front Pedia.

[4] Schlager L., Schopper M., Reinisch W. (2021). Bridging intestinal failure with teduglutide: a case report. Clin Case Rep.

[5] Müller T. (2015). Congenital secretory diarrhoea caused by activating germline mutations in GUCY2C. Gut.

[6] Varkki S. (2021). Meconium ileus due to GUCY2C gene mutations. Clin Genet.

[7] Ooms A.H.A.G. (2012). Eosinophilic myenteric ganglionitis as a cause of chronic intestinal pseudo obstruction. Virchows Arch.

[8] D’Auria E. (2021). Eosinophilic myenteric ganglionitis in a child: case report and review. Front Pedia.

